# Subendocardial reversible perfusion defects on adenosine stress MRI in ER patients with chest pain: relationship to cardiovascular risk factors

**DOI:** 10.1186/1532-429X-11-S1-P44

**Published:** 2009-01-28

**Authors:** Jan Skrok, David Dombroski, Steven M Shea, Mark Bohlman, Christine H Lorenz, Joao A Lima, David A Bluemke, Jens Vogel-Claussen

**Affiliations:** 1 Department of Radiology, Johns Hopkins University, Baltimore, MD USA; 2 Siemens Corporate Research Inc., Baltimore, MD USA; 3 Department of Cardiology, Johns Hopkins University, Baltimore, MD USA

**Keywords:** Perfusion Defect, Steady State Free Precession, Stress Perfusion, Gadopentetate Dimeglumine, Reversible Perfusion Defect

## Background

Many patients with angina-type chest pain have normal coronary angiograms and nuclear cardiac SPECT exams. This is thought to be a result of microvascular disease.

## Purpose

To evaluate patients presenting to the ER with chest pain and negative cardiac enzymes by stress perfusion MRI and to correlate the findings with future cardiovascular events over a 9 month follow-up period.

## Methods

**Figure 1:**
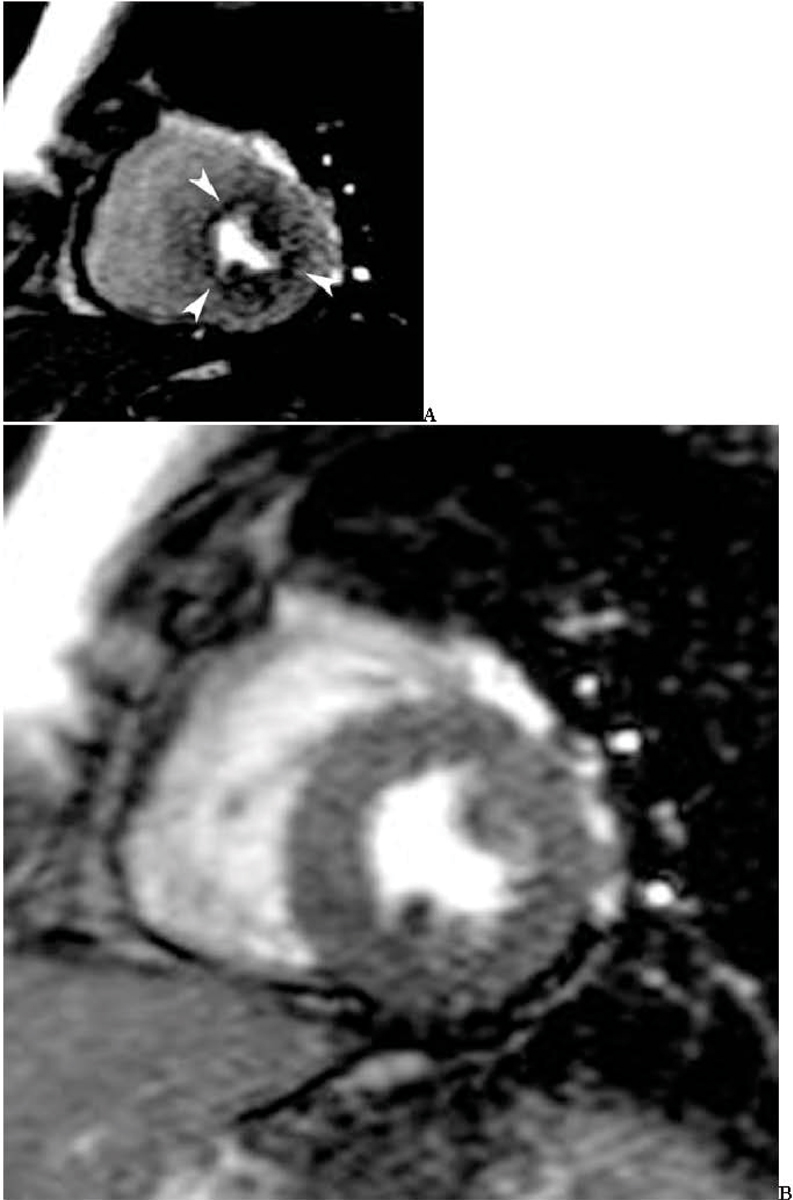
**53 year old female patient with diabetes and hypertension, presenting to the emergency room with chest pain**. (A) Diffuse subendocardial perfusion defect on adenosine stress MRI which was reversible at rest (B). The nuclear cardiac SPECT exam was normal, and there was no myocardial scar on delayed enhancement MRI.

27 patients (15 male, 12 female, mean age 56.3 ± 13.2 years) underwent a comprehensive stress cardiac adenosine MRI and a nuclear cardiac SPECT exam within 24 h after presenting to the emergency room with chest pain. MRI at 1.5 T (Siemens Avanto, Erlangen, Germany) included steady state free precession (SSFP) cine MR imaging in short and long axis planes, adenosine stress and rest perfusion imaging, as well as delayed enhancement MR imaging. For the stress perfusion MR images, adenosine was injected i.v. at a rate of 140 μg/kg/min over six minutes. Four minutes into the adenosine injection, saturation recovery (SR) SSFP MR images were obtained. Scan parameters were: TR/TE 2.4/1.0 ms, TI 180 ms, 50° flip angle, bandwidth 1000 Hz/pixel, FOV 36 × 27 cm, matrix 192 × 115, acquisition duration 150 ms, slice thickness 8 mm, acceleration factor 2 (GRAPPA). Gadopentetate dimeglumine was injected at 5 cc/sec (0.075 mmol/kg), immediately followed by a 20 cc normal saline flush at 5 cc/sec for both rest and stress perfusion. Three short axis slices and one horizontal long axis slice were acquired with a temporal resolution of 2 RR. Resting perfusion images were obtained 10 min after the stress perfusion. Two experienced investigators evaluated all images in consensus. Patients were then followed for an average of 9.1 ± 3.9 months for subsequent cardiovascular events. Figure 1.

## Results

On MRI, 8/27 patients (30%) had diffuse subendocardial perfusion defects on stress imaging in more than 1 coronary artery territory, which were reversible at rest. All of these patients had a normal SPECT cardiac stress exam and no myocardial delayed enhancement on MRI. Fifteen percent (4/27 patients) had focal reversible perfusion defects on MRI in a single coronary artery territory that correlated with ≥ 70% stenosis on conventional angiography. One patient had both small and large vessel disease (history of coronary artery bypass graft and stress induced diffuse subendocardial perfusion defects on MRI). Fifty-six percent (15/27 patients) had neither small nor large vessel disease on MRI; all were normal on SPECT and did not show any scar on MRI. Patients with diffuse perfusion defects had a significantly higher rate of diabetes (p = 0.03, two tailed Fisher's) and hypertension (p = 0.05, two tailed Fisher's) compared to patients without perfusion defects. Patients with diffuse perfusion defects had significantly more risk factors for cardiovascular disease (mean 4.4) compared to patients without diffuse subendocardial perfusion defects or significant large vessel disease (mean 2.9, p = 0.02 two-sided Wilcoxon test). Over a 9 month period, 3 patients presented again with angina-like chest pain, all of whom had shown diffuse subendocardial perfusion defects on adenosine stress MRI at baseline.

## Conclusion

Diffuse subendocardial perfusion defects on stress MRI in the emergency room setting were frequent (30%) in our patient population in the absence of positive findings on cardiac SPECT or myocardial scar on MRI. These findings were more frequent in patients with hypertension and diabetes and may relate to underlying microvascular disease. This could account for chronic recurrent chest pain in this patient population.

